# Exploring Body‐Specific Associations in Swipe Gestures: A Study on Hand Dominance and Emotional Valence

**DOI:** 10.1002/ijop.70154

**Published:** 2026-01-05

**Authors:** Marta Maisto, Silvia Serino, Marcello Gallucci, Rossana Actis‐Grosso

**Affiliations:** ^1^ Department of Psychology University of Milan‐Bicocca Milan Italy; ^2^ Bicocca Center for Applied Psychology University of Milano‐Bicocca Milan Italy; ^3^ Milan Centre for Neuroscience Milan Italy

**Keywords:** affective valence, body specificity, embodied cognition, swipe interactions

## Abstract

The Body‐Specificity Hypothesis suggests that the area around the dominant hand is perceived positively, contrasting with a negative perception around the non‐dominant hand. Given the pervasive use of interactive technologies, our study aimed to investigate body‐specific associations in real‐world settings, examining whether these are modulated by mainstream digital gestures like swiping. *N* = 30 right‐handed participants (Experiment 1) and *N* = 30 left‐handed participants (Experiment 2) were asked to make valence judgements on 28 valence‐laden images on a tablet, with each hand in separate sessions, engaging in a congruent task (swipe towards the dominant side—positive, swipe towards the non‐dominant side—negative) and an incongruent task (the opposite response pattern). Following the valence judgement task, participants assessed the intensity of their responses using a 9‐point Likert scale. Results indicated that right‐handers were faster in the congruent condition than in the incongruent condition and showed faster responses when swiping for negative images with the non‐dominant hand. Left‐handed participants did not show differences in response times but evaluated images as more positive/negative in the congruent condition compared to the incongruent. Overall, these findings support the Body‐Specificity Hypothesis and underscore the importance of considering the embodied‐cognition‐framework as susceptible to the influence of technology use.

## 1 Introduction

The Body Specificity Hypothesis (BSH) proposed by Daniel Casasanto (2009), posits that right‐handers associate ‘good’ with the dominant hand's space and ‘bad’ with the non‐dominant hand's space. In line with the embodied cognition framework, these associations arise from the ease of actions performed with the dominant hand, leading to a sense of fluency and positive affect (Beilock and Holt 2007). The BSH suggests that this link extends beyond fluency to the associated side, creating space–valence associations. This has been confirmed in a direct replication study conducted by Yamada et al. (2024), in various cultures (de la Fuente et al. 2015), in job interview simulations, dating decisions (Casasanto 2009), and even politicians' gestures during speeches (Casasanto and Jasmin 2010). In addition, in response time paradigms involving lexical decision tasks (e.g., de la Vega et al. 2015), hand compatibility effects were found: specifically, right‐handed participants were faster for positive words when using their dominant hand to press a key on the right side of the keyboard and for negative words when using their non‐dominant hand to press a key on the left side of the keyboard. This result was also found in left‐handed participants, who showed the opposite pattern of behaviour compared to right‐handers.

de la Vega et al. (2013) questioned whether the origin of the BSH is extracorporeal (space‐valence associations) or intracorporeal (hand‐valence associations). Their study allowed for a clear distinction between these possibilities by asking participants to press the left keys with their right hand and right keys with their left hand, thereby eliminating factors confounded in the preceding studies. The results highlighted the intracorporeal origin of the BSH, indicating that hand‐valence associations override space‐valence associations.

Furthermore, lateralized movements on a keyboard can also influence valence evaluation: right‐handed individuals tend to evaluate neutral words more positively after fluent rightward movements and more negatively after non‐fluent leftward movements, reinforcing the association of positivity with the right and negativity with the left (Milhau et al. 2013). This effect, referred to as the valence reinforcement effect, has also been found on a large touchscreen during a grab‐and‐drag interaction with digital pictures (Cervera Torres et al. 2020). Specifically, in the study by Cervera Torres et al. (2020) right‐handed participants evaluated the emotional valence of positive and negative pictures after interacting with them using either the dominant (right) or non‐dominant (left) hand. In this study, pictures were moved laterally on a large touchscreen, either from left to right or right to left or vice versa. Interestingly, in the interactive environment used by Cervera Torres et al. (2020), a valence‐reinforcement effect emerged: positive images were rated more positively only when participants interacted with them using their dominant (right) hand on the right side, whereas negative images were rated more negatively when interacted with using their non‐dominant (left) hand on the left side. Thus, although the findings align with the BSH, the reinforcement effect depended not only on which hand was used but also on the spatial characteristics of the interaction: in other words, the combination of the hand used, the image's valence, and the direction of movement amplified the initial emotional appraisal. In this vein, a follow‐up study (Torres et al. 2024), focusing on RTs, used only the grab part showed that lateral embodied interactions with affective digital content—such as performing a ‘grab’ gesture on emotional images—elicit an interactive positivity bias, with participants demonstrating significantly faster responses when grabbing positive images on the right side compared to the opposite scenario.

Interestingly, this kind of interactive settings based on, for instance, grab and drag interactions with digital objects (e.g., Brucker et al. 2021; Cervera Torres et al. 2020, 2021) is grounded in the ‘Embodied Interaction’ (EI, Dourish 2001) framework, which emphasises the role of interactive technologies in embodied cognition. Similarly, evidence shows that vertically embodied interactions via swipe gestures on a touchscreen, executed either towards or away from the body, reinforce the perceived positivity of images and mitigate the perceived negativity, as opposed to mere passive observation (Cervera‐Torres et al. 2021).

Prior work (e.g., Cervera Torres et al. 2020) presents several limitations that motivate the present study. First, their paradigm relied on a grab‐and‐drag sequence of actions, which may conflate distinct action components (e.g., reach, grasp, drag). Second, they recruited only right‐handed participants. Third, data were collected on large touchscreens, a context that requires crossing the body midline. Fourth, lateral swipe gestures, now ubiquitous on smaller devices and apps, were not investigated. Addressing these gaps is essential to determine whether and how interaction design and common mobile gestures shape space–valence associations in realistic mobile contexts.

Therefore, this study examines the extent to which lateralized swipe interactions on touch‐based interfaces may affect body‐specific associations during valence judgements. The choice of swipe interactions is driven by several considerations. First, these interactions occur on touch‐based interfaces—such as smartphones, tablets and touch monitors—that support interactive gestures that engage the fingers, hands and arms replicating the manual interactions typically performed with tangible objects (Fabbri et al. 2017).

Secondly, the use of tablet interfaces allows us to advance the understanding of embodied interactions (i.e., space–valence associations) considering the ease of interaction (e.g., Wigdor and Wixon 2011).

Moreover, it's important to underline that many mobile apps are designed such that a leftward swipe signals rejection (i.e., negative evaluation) while a rightward swipe denotes interest (i.e., positive evaluation). For example, in the Tinder app a right‐swipe ‘likes’ a profile and a left‐swipe dismisses. This observation raises the question of whether body‐specific associations are influenced by such design, potentially causing left‐handed individuals to exhibit right‐handed preferences. Additionally, the role of active movement is critical, as it can reinforce body‐specific associations.

## 2 Aim and Research Questions

Our primary aim was to evaluate whether a specific interaction design—based on lateralized swipe gestures with affective stimuli—yields behavioural (response time, RT) and explicit valence evaluations consistent with the BSH in both right‐ and left‐handers. We further investigated whether common mobile app conventions (right‐swipe positive, left‐swipe negative) might override natural body‐specific associations in left‐handers, inducing a right‐hand bias in their affective responses. To test this, we directly compared left‐ and right‐handed participants using a tablet‐based paradigm in which users executed lateralized swipes from the center towards the right or left. In this way, we extended and refined the study by Cervera Torres et al. (2020), which included only right‐handed participants. Our paradigm isolates the influence of touch‐based interactions on body‐specific associations: by comparing response patterns in left‐ and right‐handers, we can determine whether these associations arise from embodied factors rather than from habitual mobile‐app swipe conventions. Furthermore, we believe that our setup more accurately reflects the everyday interactions typical of mobile touch‐based devices.

Additionally, we included RT as an additional dependent variable alongside valence evaluation, enabling a more comprehensive analysis of behavioural responses.

To this aim, right‐handers (Experiment 1) and left‐handers (Experiment 2) were asked to perform a valence judgement task through a lateral swipe interaction that could be either congruent or incongruent with their space–valence associations with one hand at a time.

We predicted two possible patterns:

1. In line with the BSH, positive images would be perceived as more positive and negative images as more negative in the congruent condition compared to the incongruent condition in both experiments (H1). Regarding RT and space‐valence associations, we predicted that right‐handed participants would respond faster when swiping right for positive images and left for negative images (H2), while left‐handed participants would respond faster when swiping left for positive images and right for negative images (H3). For hand‐valence associations, we hypothesised that both handedness groups would respond faster to positive stimuli with their dominant hand and to negative stimuli with their non‐dominant hand (H4). The presence of both space‐valence and hand‐valence associations would indicate a dual mechanism. If only space‐valence associations emerge, this would suggest an extracorporeal origin of BSH; if only hand‐valence associations emerge, this would suggest an intracorporeal origin.
2. Alternatively, left‐handed participants might show right‐handed patterns having adapted to the prevailing mobile‐app design in which a rightward swipe denotes interest and a leftward swipe signifies rejection.

## 3 Experimental Design

To test these hypotheses, we conducted two experiments: one with right‐handers (Experiment 1) and one with left‐handers (Experiment 2). Each experiment employed a 2 × 2 × 2 within‐subjects design, with the experimental condition (congruent vs. incongruent), hand (right vs. left), and valence category of stimuli (positive vs. negative) as factors.

Ethical approval for the study was granted by the Committee for Research Evaluation of the University of Milan‐Bicocca (Protocol number RM.2022‐544).

## 4 Experiment 1

### 4.1 Method

#### 4.1.1 Participants

A total of 30 healthy right‐handed participants (22 females, *M*_age_ = 27.2, SD_age_ = 2,6) from the University of Milan‐Bicocca took part in Experiment I as volunteers. All participants had normal or corrected to‐normal vision and were naive to the purpose of the experiment. The sample size was established based on the power analysis conducted via Monte Carlo simulations, using the *simr* R package (Green and MacLeod 2016). A dataset with N cases and a 2 × 2 × 2 design repeated 8 times per case was built for each simulation. The target effect size, expressed as a Cohen's *d*, was set for a 2 × 2 interaction according to Judd et al. (2012) and was set to 0.40, a small to intermediate value (Cohen 1977). The rest of the model was set with intercepts and the target interaction slope as random effects across cases. This guarantees the correct degrees of freedom and standard errors in the simulations. A series of simulations was run changing the number of cases until the required power was attained. For each *N*, a set of 1000 simulations was run. Results show that a sample of *N* = 30 participants yields an expected power of 0.95 for *d* = 0.40, and that the sample guarantees a detection with power equal to 0.80 for effect sizes as small as *d* = 0.30.

#### 4.1.2 Apparatus and Stimuli

A Samsung Galaxy Tab A8 served as the display device for presenting stimuli. The touchscreen monitor (TM) on the tablet measured 10.5 in. with a resolution of 1920 × 1200 pixels. The experiment was conducted using a native Android mobile app created by the BiCApP (Bicocca Center for Applied Psychology). This app, integrated with Qualtrics (https://www.qualtrics.com/), automatically recorded all users' session data in a non‐interactive Qualtrics survey.

Stimuli were 80 images, all of uniform size and resolution (640 × 426), sourced from the high‐quality, royalty‐free image website ‘Pixabay’ (https://pixabay.com/it/). Importantly, the images were deliberately chosen with the specific objective of including both positive and negative content. This careful selection resulted in 40 positive images and 40 negative images for evaluation, avoiding images portraying human beings. Following this initial stimulus selection, we conducted a manipulation check procedure to ensure that our stimuli accurately represented our target valence‐laden images.

#### 4.1.3 Stimuli Validation

A manipulation check was conducted to validate stimuli before primary data collection. Sixty participants (34 females, *M*_age_ = 26.2, SD_age_ = 3.6), different from those in the experimental protocol, were tested individually on a laptop. They completed a valence judgement task for images displayed on a 15.6‐in. monitor (1366 × 768 pixels). Participants clicked a box to indicate whether each image was positive or negative, then rated the intensity of their response on a Likert scale from 1 (‘poorly positive/negative’) to 5 (‘very positive/negative’).

Results showed that 68% of positive images (41 out of 60) and 47% of negative images (28 out of 60) were rated as intended by at least 80% of participants.

We selected images that received unanimous evaluations (100%) with scores from 4 to 5, resulting in 28 images (14 positive, 14 negative) for the experimental phase. This ensured that each image's valence aligned with our testing parameters. Selected images and their descriptive statistics (mean and std. dev.) are available in the Open Science Framework (OSF) repository.

#### 4.1.4 Procedure

**FIGURE 1:**
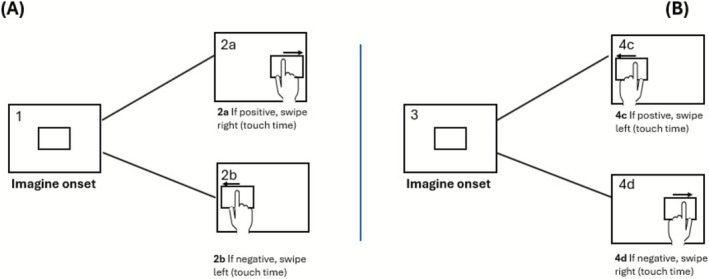
Participants underwent the *Congruent block* (Panel A) in which, after the image onset (1), they swiped right for positive images (2a) and left for negative images (2b) and the *Incongruent block* (Panel B) where, after the image onset (3), they swiped left for positive images (4c) and right for negative image (4d).

Participants were individually tested in a valence judgement task, evaluating both positive and negative images across two sessions. They sat at a desk with a tablet centred in front of them, displaying a Native Android mobile app. Participants were informed they could interrupt the experiment anytime and that they would encounter two experimental blocks without being informed of their nature (congruent and incongruent), which they would repeat with the other hand after 48 h (hand order was counterbalanced). Breaks of about 10 min between conditions were allowed if needed. The study's aim and procedure were explained, and all participants read and signed a consent form before data collection in accordance with the Declaration of Helsinki (World Medical Association 2013). Handedness was tested with the Edinburgh Inventory (Oldfield 1971), in Italian translation; additionally, we also included three ad hoc items to screen and include participants who used mobile devices and applications on a daily basis. Each task began with a brief training phase and verbal instructions using two images. Participants judged whether an image was positive or negative by swiping accordingly. In the Congruent block, participants swiped right for positive stimuli and left for negative stimuli. In the Incongruent block, the swiping direction was reversed (Figure 1). Instructions clarified the need to reverse the swipe direction between blocks. The order of blocks was counterbalanced to mitigate bias.

**FIGURE 2:**
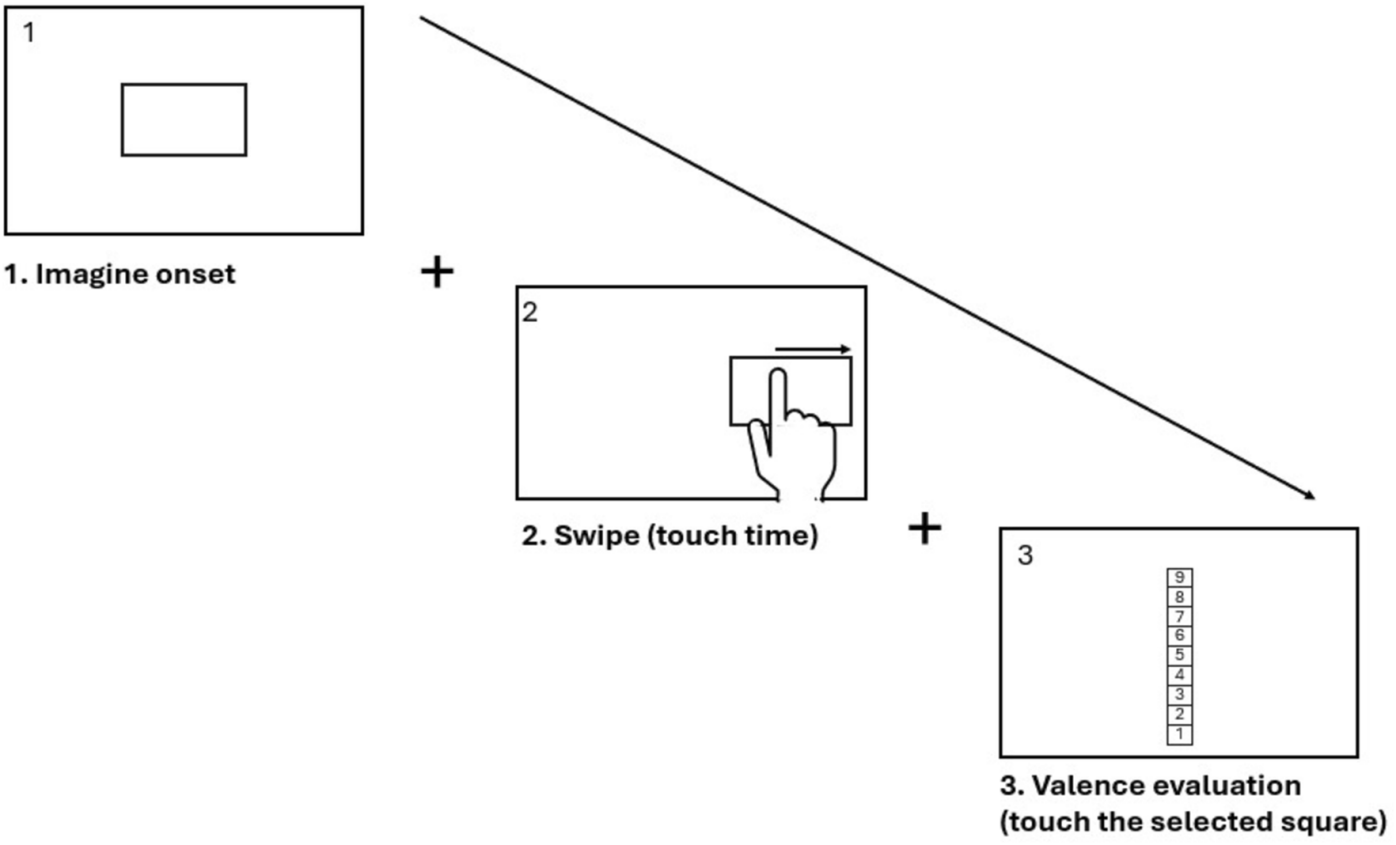
Schematic representation of the procedure. The task began with the presentation of an image (Panel 1). Participants then swiped in the direction corresponding to their valence judgement (Panel 2). Finally, they rated the intensity of their emotional response to the image on a 9‐point Likert scale, ranging from 1 (*slightly positive/negative*) to 9 (*highly positive/negative*) by touching the corresponding square (Panel 3).

After each valence judgement, the picture disappeared, and participants rated the intensity of their response on a Likert scale from 1 (*poorly positive/negative*) to 9 (*very positive/negative*) by touching the corresponding square (Figure 2). A countdown from 3 to 1 preceded the presentation of each new image. Each session lasted about 10 min. Response time (RT) was measured from image onset to the participant's swipe. Valence evaluation (VE) and RT served as dependent variables. Each participant completed 112 trials (14 positive and 14 negative images × 2 hands × 2 conditions).

### 4.2 Statistical Analysis

Data were analysed using two linear mixed effects models (LMM), one for RTs and one for VEs, with the software Jamovi, version 2.5.6 with the GAMLj module version 3.5.1. (Gallucci 2024). All statistical tests were conducted on a two‐tailed 0.05 level of significance. To calculate *p* value estimates for the fixed effects, it was used a Type III Satterthwaite approximation (e.g., Carr et al. 2016). All models had Condition (congruent vs. incongruent), Hand (right vs. left), and Valence (positive vs. negative) and their interactions as fixed effects. Regarding the model considering RTs we transformed the dependent variable (RT) using a logarithmic function due to its significant skewness. To select an appropriate random component, we started setting all plausible effects as random. The converging model had random intercepts, and random slopes for Valence, Hand and Condition. For RTs, only response times corresponding to correct answers were preserved in the statistical analysis, leading to the exclusion of 13% of data. RTs greater or less than 2SDs of the subject average in each experimental group were excluded from final analyses (7%).

Regarding VE model, the one that converged included the random intercepts across participants and the effects (slopes) of the experimental Condition and Valence as random coefficients across participants.

### 4.3 Results

#### 4.3.1 Response Time

**FIGURE 3:**
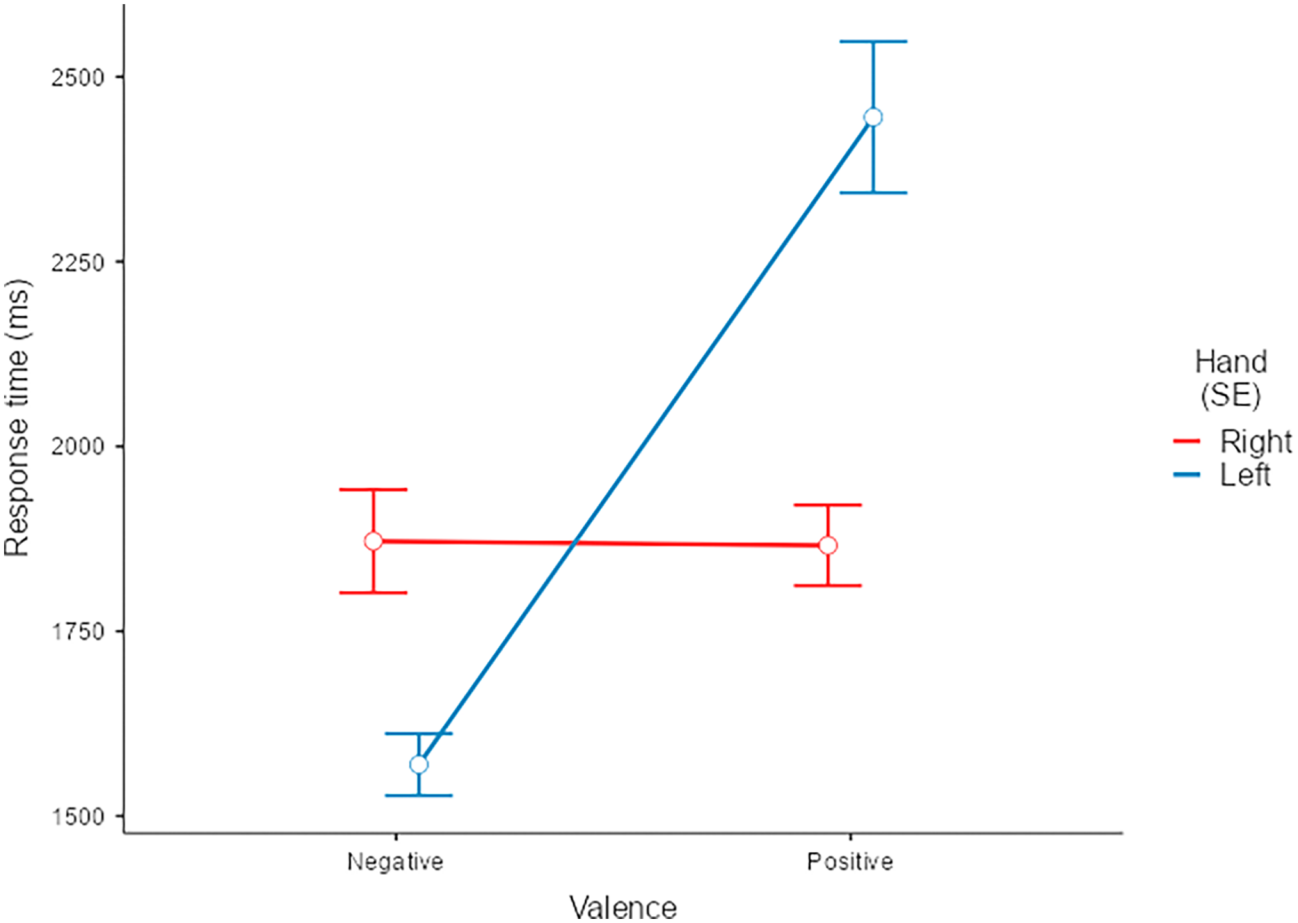
RTs as a function of Valence and Hand. RTs are lower for the non‐dominant left hand when interacting with negative stimuli compared with positive ones (*p* < 0.001). Error bars indicating Standard Errors (SE).

A main effect of Condition *F*(1,23) = 8.01, *p < 0*.01 showed that the experimental condition had a significant influence on participants' swiping behaviour. Specifically, right‐handed participants were faster in the Congruent condition (CC) (*M* = 1851, SE = 40.8, C.I. = [1767,1935]) than in the Incongruent condition (IC) (*M* = 2026, SE = 72.7, C.I. = [1875,2176]). Interestingly, a significant interaction between Hand and Valence emerged *F*(1,27) = 148.62, *p* < 0.001 (Figure 3, Table 1 for means and confidence intervals). Specifically, right‐handed individuals, when swiping with the non‐dominant left hand, were faster for negative images than for positive images, *F*(1,23.3) = 176.56, *p* < 0.001, whereas when swiping with the dominant right hand, no difference in RT emerged, *F*(1,26.7) = 0.002, *p* = 0.96. However, simple effects analyses directly comparing the two hands revealed that the left hand was faster than the right for negative images, *F*(1,26.1) = 37.1, *p* < 0.001, whereas the right hand was faster than the left for positive images, *F*(1,23.1) = 47.5, *p* < 0.001. This pattern is consistent with BSH. The interactions between Valence and Condition, *F*(1,1436) = 0.06, *p* = 0.79, and Hand and Condition, *F*(1,436) = 2.94, *p* = 0.08 were not significant, as well as the three‐way interaction, indicating that the combined influence of these factors did not significantly affect participants' responses. Overall, the model explained a good part of variance (R_c_^2^ = 0.450, R_m_^2^ = 0.245).

**TABLE 1:** RT: estimate marginal means—valence*hand.

					95% confidence intervals	
Valence	Hand	Mean	SE	df	Lower	Upper
Negative	Right	1872	69.7	23.4	1728	2016
Negative	Left	1570	41.7	41.8	1485	1654
Positive	Right	1866	54.7	24.8	1754	1979
Positive	Left	2445	102.1	23.0	2234	2657

#### 4.3.2 Valence Evaluation

A main effect of Valence *F*(1,23) = 9,74, *p* < 0.01 was found with higher evaluation for negative images (*M* = 7.57) than positive images (*M* = 7.14). We found no significant effects for the two‐way interactions between Valence and Hand, *F*(1,2634) = 0.54, *p* < 0.46 and Valence and Condition *F*(1,2634) = 0,01, *p* = 0.89. However, there was a marginally‐significant interaction effect between Condition and Hand, *F*(1,2611) = 3.16, *p* = 0.07 with lower VE for the right hand in the CC (*M* = 7.31) compared to the IC (*M* = 7.41), and higher VE for the left hand in the CC (*M* = 7.39) compared to the IC (*M* = 7.31). This suggests a nuanced relationship between the choice of hand and the experimental condition, warranting further investigation. Overall, the model explained a good part of variance (R_c_^2^ = 0.318, R_m_^2^ = 0.019).

### 4.4 Discussion

In Experiment 1, we hypothesised faster responses for both positive and negative images in the congruent swipe pattern compared to the incongruent pattern. Results confirmed these predictions, showing quicker evaluations in the congruent pattern and slower in the incongruent pattern, reinforcing the BSH's extracorporeal origin (Casasanto 2009).

Interestingly, our study also revealed a strong hand‐compatibility effect. Specifically, responses with the left hand were faster than the right for negative images, whereas responses with the right hand were faster than the left for positive images. This pattern is consistent with the predictions of the BSH. Contrary to expectations, we did not observe either an interaction between hand and side as shown by Milhau et al. (2015), nor the valence reinforcement effect observed by Cervera Torres et al. (2020).

In summary, these findings highlight interaction effects between valence and space in unimanual swipe interactions, as well as between hand and stimuli (hand‐compatibility effects). The observed space–valence associations align with expectations for right‐handers, reflecting the prevalent mobile app design.

## 5 Experiment 2

### 5.1 Introduction

In Experiment 1, performance differences were revealed in a valence judgement task based on the congruency of responses through a swipe interaction. The key finding highlighted that right‐handers demonstrated an implicit preference for swiping right for positive images and swiping left for negative ones (congruent condition) compared to the opposite pattern (incongruent condition). This response pattern aligns both with the natural space–valence associations of right‐handed individuals and with the design of conventional mobile apps.

The intriguing question now revolves around left‐handed individuals. Will they exhibit similar behaviour to right‐handed participants? Or, alternatively, will their natural space–valence associations come into play? To address this, we conducted a replication of Experiment 1 with left‐handers.

### 5.2 Method

#### 5.2.1 Participants

A total of 30 left‐handed participants (13 females, M_age_ = 26.6, SD_age_ = 3,01) from the University of Milan‐Bicocca took part in Experiment 2 as volunteers. As for Experiment I, the sample size was established based on the power analysis conducted via Monte Carlo simulations (Green and MacLeod 2016), using *simr* R package –for the entire procedure see Experiment 1. The design, apparatus, stimuli and procedure mirrored that of Experiment 1.

### 5.3 Statistical Analysis

The data were analysed using two linear mixed‐effects models (LMM) in Jamovi, version 2.5.6 with the GAMLj module version 3.5.1. (Gallucci 2024). All models had Condition (congruent vs. incongruent), Hand (right vs. left) and Valence (positive vs. negative) and their interactions as fixed effects. Mirroring procedure of Experiment 1, we transformed the RT variable using a logarithmic function due to its significant skewness. For RT data, we initially considered all plausible effects as random components. The model that converged included the intercept, the effect of Valence, Condition, Hand and Condition by Hand interaction as random coefficients across participants. Only RT data corresponding to correct answers were retained for statistical analysis, resulting in the exclusion of 5% of the data. Additionally, RT values greater or less than 2 standard deviations (SDs) from the subject average in each experimental group were excluded from further analyses, accounting for 8% of the data.

For the valence evaluation (VE) dependent variable, a model with the intercept, Valence and Conditions as random coefficients across participants successfully converged.

### 5.4 Results

#### 5.4.1 Response Time

A significant main effect of Valence was found, indicating lower RTs for positive images (*M* = 2075) compared with negative ones (*M* = 2165), *F*(1,25) = 8.11, *p* < 0.01. Regardless of the experimental conditions, a significant main effect of Hand emerged, *F*(1,25) = 21.03, *p <* 0.001, with lower RTs for the dominant left hand (*M* = 1968) and higher RTs for the right hand (*M* = 2272). The analysis also revealed a non‐significant effect for the Condition factor, *F*(1,25) = 1.53, *p* = 0.22, suggesting that differences between experimental conditions did not significantly contribute to the observed variability. Both the two‐way interactions between Valence and Condition, *F*(1,2779) = 1.46, *p =* 0.22 and Valence and Hand *F*(1,2779) = 2.10, *p* = 0.14, were not significant, as well as the three‐way interaction. Overall, the variance explained by the model was R_c_^2^ = 0.408, R_m_^2^ = 0.043.

#### 5.4.2 Valence Evaluation

**FIGURE 4:**
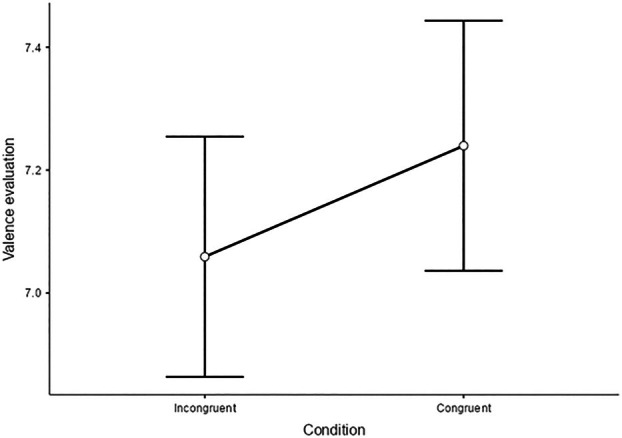
VE as a function of Conditions with higher VE in the Congruent condition compared to the Incongruent one (*p* = 0.01). Error bars indicating Standard Errors (SE).

A significant main effect of Condition was found, indicating higher evaluation for both negative and positive images in the CC compared with the IC, *F*(1,25) =7.41, *p = 0*.01, (Figure 4, Table 2 for means and confidence intervals). No statistically significant main effects were observed for Hand *F*(1,2829) = 0.09, *p* = 0.76 or Valence *F*(1,25) = 0.03, *p* = 0.86. The interaction between Valence and Hand was also not statistically significant *F*(1,2829) = 0.71, *p* = 0.39. The interaction between Valence and Condition was similarly not significant *F*(1,2829) = 1.52, *p* = 0.21. Likewise, the interaction between Hand and Condition was not significant, *F*(1, 2879) = 1.12, *p* = 0.28 as well as the three‐way interaction. Overall, the variance explained by the model was R_c_^2^ = 0.336, R_m_^2^ = 0.003.

**TABLE 2:** VE: estimate marginal means—condition.

				95% confidence intervals	
Condition	Mean	SE	df	Lower	Upper
Incongruent	7.06	0.195	25.0	6.66	7.46
Congruent	7.24	0.203	25.0	6.82	7.66

### 5.5 Discussion

The results of Experiment 2 exhibit considerable divergence compared to those of Experiment 1.^1^ Specifically, concerning our RTs data we do not observe any implicit effects associated with BSH, as neither hand‐valence associations nor valence‐space associations emerge. However, when VE data are considered, we did observe a valence reinforcement effect, where participants consistently rated more intensively positive and negative images in the congruent condition as compared to the incongruent condition. Taken together, these findings show that the BSH manifests in our left‐handed participants only at the explicit level, namely during explicit valence evaluations which align with natural left‐handers' space valence associations.

We hypothesize that the absence of effect for RTs may be attributed to left‐handers' habitual exposure to environments—both physical and digital—that are typically designed for right‐handed users, including most mobile applications. Indeed, technology and product design (e.g., computer mouse, number pads, smartphone interfaces) frequently favour right‐handed users. Nevertheless, left‐handed users are generally accustomed to using such devices in a right‐handed manner (see Thomas et al. 2019).

These results are indeed consistent with the suggestion by Milhau et al. (2015), according to which left‐handers may not develop negative associations with conditions incongruent with their preferences (left‐positive, right‐negative), possibly due to their habit of encountering incongruent situations with their dominant hand. Finally, we hypothesise that this would make them more flexible, and this flexibility seems apparent in our RT data, while their preferences remain in VE data.

## 6 General Discussion

This study explores the potential influence of lateralized swipe interactions on touch‐based interfaces in shaping body‐specific associations (space‐valence, hand‐valence and valence reinforcement effects) during valence judgements.

In Experiments 1 and 2, right‐handed and left‐handed participants were instructed to swipe right for positive images and left for negative ones, and vice versa, in separate conditions. The direction of the swipe responses was manipulated to be congruent or incongruent with participants' space–valence associations, creating two opposite response patterns for right‐handers and left‐handers.

Our expected results could either be aligned with the BSH (Casasanto 2009) or reflect the influence of swipe conventions commonly used in mobile apps (e.g., Tinder app). This means that if the first case proved to be true, we should observe two opposite patterns of responses for right and left‐handers; whereas, if the second was the case, the same pattern should be observed in both populations.

Our results indicated that regarding RT data, right‐handed participants' responses aligned with the BSH: they were faster in swiping images congruently with their space‐valence associations using both their dominant and non‐dominant hands. In contrast, left‐hander individuals' responses aligned with BSH only at the explicit level when VE is considered. Taken together, these results suggest that for left‐handers, the BSH effect (present explicitly) might be modulated at the implicit level by the extensive use of mobile apps, leading to a levelling of the effect. This result is particularly interesting given that it became apparent in an ecological setting where the required movement was the same as the one usually used in mobile apps (i.e., swipe gesture). In other words, the usual action of swiping right for positive and left for negative might have cancelled out the natural body‐specific association for left‐handers, which has the opposite pattern (left for positive, right for negative). In this perspective, the interactive setting we used enabled a better understanding of the flexibility of body‐specific effects already demonstrated by Casasanto and Chrysikou (2011). The swiping response pattern in fact induced a modulation of the effect (only at an explicit level), while in Casasanto and Chrysikou (2011) the modified fluency of the dominant hand induced a reversed effect. Indeed, contextual factors, such as standard app‐use conventions, could modify the ‘natural’ space–valence associations. For example, a momentaneous space‐valence ‘alteration’ has been empirically demonstrated by Casasanto and Chrysikou (2011). In their study, participants' dominant‐hand fluency was experimentally altered, which led to a reversal of the typical body‐specific effects. Similarly, our study showed that the continuous swiping response pattern used in common mobile apps might have, over time, influenced left‐handers' responses, suggesting that interaction context might implicitly modulate space–valence associations. In this vein, our results are consistent with the CORE principle proposed by Pitt and Casasanto (2022), which suggests that spatial metaphors are shaped by cultural, ordinal and recurrent experiences. This perspective implies that the frequent right‐for‐positive/left‐for‐negative swipe pattern could contribute to modifying the ‘natural’ space valence associations, particularly for left‐handed users.

Additionally, another noteworthy effect observed in right‐handed participants was the hand compatibility effect. Specifically, right‐handed individuals showed faster responses to negative images with their left hand compared to their right hand. Strikingly, this type of hand compatibility effect was not observed in left‐handed participants.

In this vein, complementary work on hand‐proximity effects demonstrates that bringing the hand near a stimulus facilitates its processing (e.g., Festman et al. 2013). In line with this argument, it might be plausible that increased salience of stimuli when hands are near them may have strengthened the match between an image's emotional valence and its spatial ‘meaning’ (e.g., positive images in the right‐hand ‘approach’ zone; negative images in the left‐hand ‘avoidance’ zone). This account is supported by Cervera‐Torres et al. (2021), who reported that hand proximity enhanced perceived pleasantness only when interactions followed natural approach–avoidance patterns—moving pleasant images closer and unpleasant images farther away.

In addition, our study fits nicely with the EI theoretical framework (Dourish 2001) and provides an important contribution by investigating how direct interaction with digital objects shapes emotional meaning.

Importantly, this study offers insights grounded in real‐world scenarios, providing a valuable perspective within ecological settings. Firstly, the exploration of space–valence associations, traditionally confined to experimental tasks such as preference tasks (e.g., Casasanto 2009), speech analysis (Casasanto and Jasmin 2010), and response time paradigms (de la Vega et al. 2012, 2013; Milhau et al. 2013, 2015), has been extended to interactive environments. Specifically, our results showed that space–valence associations are observable in tablet‐based interfaces via swipe gestures on mobile devices, demonstrating that these effects persist and can be modulated in more technology‐mediated contexts. Secondly, the swipe interaction plays a crucial role with its distinct set of mechanics and meanings.

## 7 Limitations and Future Directions

While our stimuli were carefully controlled and rated for valence, they were not matched on arousal or luminance. Future research should examine how discrete emotional categories (e.g., anger, fear, joy) interact with hand‐ and space‐valence associations. It will also be important to test whether these effects extend to more diverse populations—such as children or non‐technology‐expert users—to provide a robust countercheck of our findings, particularly among left‐handers. We are currently designing follow‐up experiments to address these questions in greater depth.

## 8 Conclusion

This study shows that the BSH remains relevant involving swiping interactions, albeit exhibiting variations compared to outcomes observed via traditional experimental tasks. In particular, it is shown to be modulated by daily mobile apps use.

## Author Contributions

**Marta Maisto:** conceptualization, methodology, writing – original draft, formal analysis, writing – review and editing. **Silvia Serino:** methodology, review and editing. **Marcello Gallucci:** formal analysis, supervision. **Rossana Actis‐Grosso:** conceptualization, methodology, writing – review and editing, supervision.

## Funding

This work was supported by a Doctoral scholarship from the University of Milan‐Bicocca to Marta Maisto.

## Ethics Statement

All procedures performed in studies involving human participants were in accordance with the ethical standards of the institutional research committee at the University of Milan‐Bicocca and with the 1964 Helsinki Declaration and its later amendments or comparable ethical standards.

## Consent

Informed consent was obtained from all individual participants involved in the study.

## Conflicts of Interest

The authors declare no conflicts of interest.

## Supporting information


**Data S1:** ijop70154‐sup‐0001‐Supinfo.pdf.

**Data S2:** ijop70154‐sup‐0002‐Supplementarytable.pdf.

## Endnotes

^1^
Full list of results and statistical comparison between Experiment 1 (right‐handers) and Experiment 2 (left‐handers) is reported as Supporting Information (available at https://osf.io/72hps/overview?view_only=d30f3a3d7cfb414b972771235ca2a33b).
