# Supplementary material for: Exploring Body‐Specific Associations in Swipe Gestures: A Study on Hand Dominance and Emotional Valence
Source: Int J Psychol. 2026 Jan 5;61(1):e70154. doi: 10.1002/ijop.70154 (PMC12770064; doi:10.1002/ijop.70154)
Supplement: Supplementary file 1 — Data S1: ijop70154‐sup‐0001‐Supinfo.pdf. [file IJOP-61-e70154-s002.pdf]

Supplementary material

1. **Statistical comparison of the effects in Experiment 1 and Experiment 2**

To compare the results obtained by the right-hand sample and the left-hand sample, we conducted a combined analysis. We first merged the two datasets and introduced **Handedness** (right- vs. left-handers) as an additional factor. Data were analyzed using two linear mixed effects models (LMM), one for RTs and one for VEs, with the software Jamovi, version 2.5.6, (*The Jamovi Project*, 2024), with the GAMLj module version 3.5.1. (Gallucci, 2024). To select an appropriate random component, we started setting all plausible effects as random. The model with the intercept, valence, hand, condition, and the hand x condition interaction as random coefficients was the one that converged. Thus, the final model included random intercepts across participants, and the effects (slopes) of the experimental Condition (congruent vs incongruent), Hand (right vs left), Valence (positive vs negative), Hand x Condition interaction as random coefficients across participants and Handedness (left-handers vs right-handers) as fixed factor. The effects were compared by examining the interactions between Handedness and the experimental factors.
To calculate p value estimates for the fixed effects, it was used a Type III Satterthwaite approximation (e.g., Carr et al., 2016). Regarding VE model, the one that converged included the intercept, condition, handedness as random coefficients along with their interaction, with hand and valence as fixed factors.

Results

Valence evaluation

The **Condition × Handedness interaction** was statistically significant $F(1,47) = 4.15$, $p = .04$, indicating that left-handed participants, compared with right-handed, evaluated as more negative and as more positive images in the Congruent Condition compared with the Incongruent Condition (Figure SM1)
The **Valence × Handedness interaction** was significant too $(1,5487) = 26.29$, $p < .001$ indicating that right-handed participants, compared with left-handed made higher evaluation for negative images compared with positive ones (Figure SM2).
The Handedness × Hand interaction was not significant, $F(1, 5487) = .09$, $p = .75$. Likewise, neither of the three-way interactions—Condition × Handedness × Valence nor Condition × Handedness × Hand—nor the four-way interaction among Condition, Handedness, Valence, and Hand reached significance (all $p \geq .05$).

Effects Plots

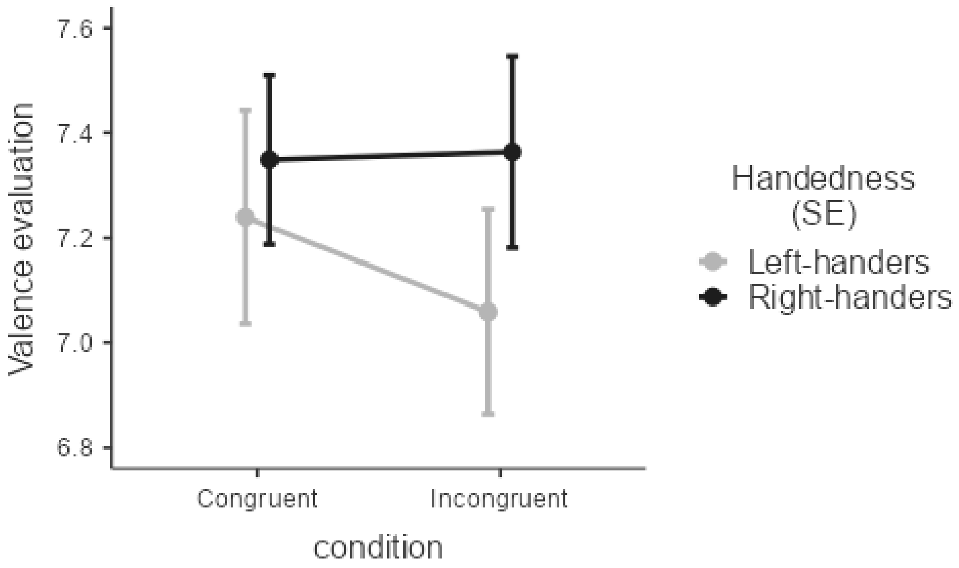

Figure SM 1 . VEs as a function of Condition for the left-handed group. VEs are higher in the Congruent condition compared with the Congruent Condition (p =.04). Error bars indicate Standard Errors (SE).

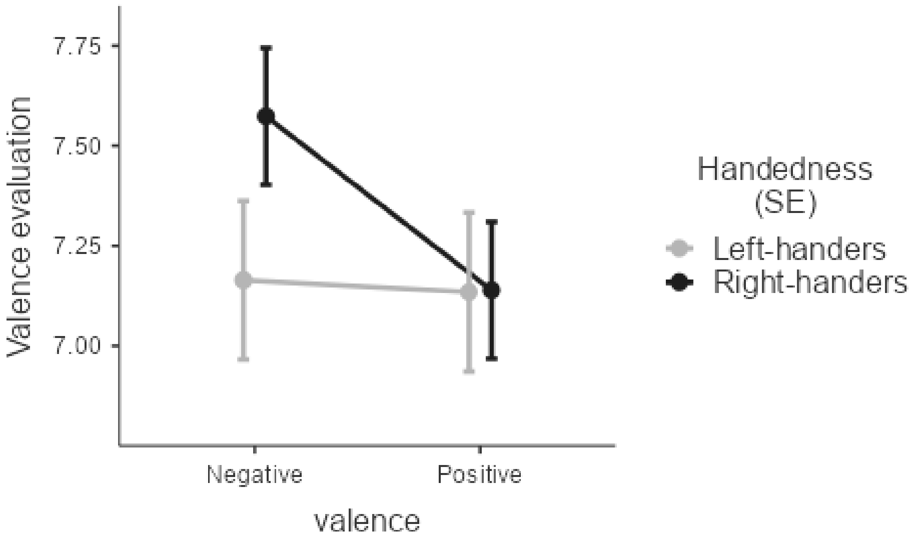

Figure SM2. VEs as a function of Valence for the right-handed group. Ves are higher for negative images compared with positive ones (p <.00). Error bars indicate Standard Errors (SE).

These combined results for VE correspond with those observed in the two individual experiments: We found significant interactions with Handedness for the effects that were interpreted as different in the two groups in the main text and non-significant interactions for effects that were interpreted as equivalent in the two groups.

Response time

The **Condition × Handedness interaction** was not statistically significant , $F(1,96) = 1.19$, $p = .27$ showing that the two groups' effects were not remarkably different. However, in line with the main text results, the effect of Condition was significant for right-handed participants (Simple effect, $F(1,68) = 6.39$, $p = .01$) and not significant for left-handed participants (Simple effect, $F(1,58) = 1.52$, $p = .22$).

The **Handedness x Hand interaction** was statistically significant $F(1,74) = 15.81$, $p < .001$ showing faster responses with the dominant hand for both right-handed and left-handed participants (Figure SM3).

The three-way **Handedness x Valence x Hand** interaction was statistically significant $F(1,59) = 96.46$, $p < .001$ showing that the interaction between Valence and Hand is different across dominance domain. In line with the results of the main text the Simple Interactions Valence x Hand is significant for right-handed participants $F(1,73) = 179.35$, $p < .001$ but not left-handed ones $F(1,35) = 1.42$, $p = .24$ (see Figure SM4).

Effects Plots

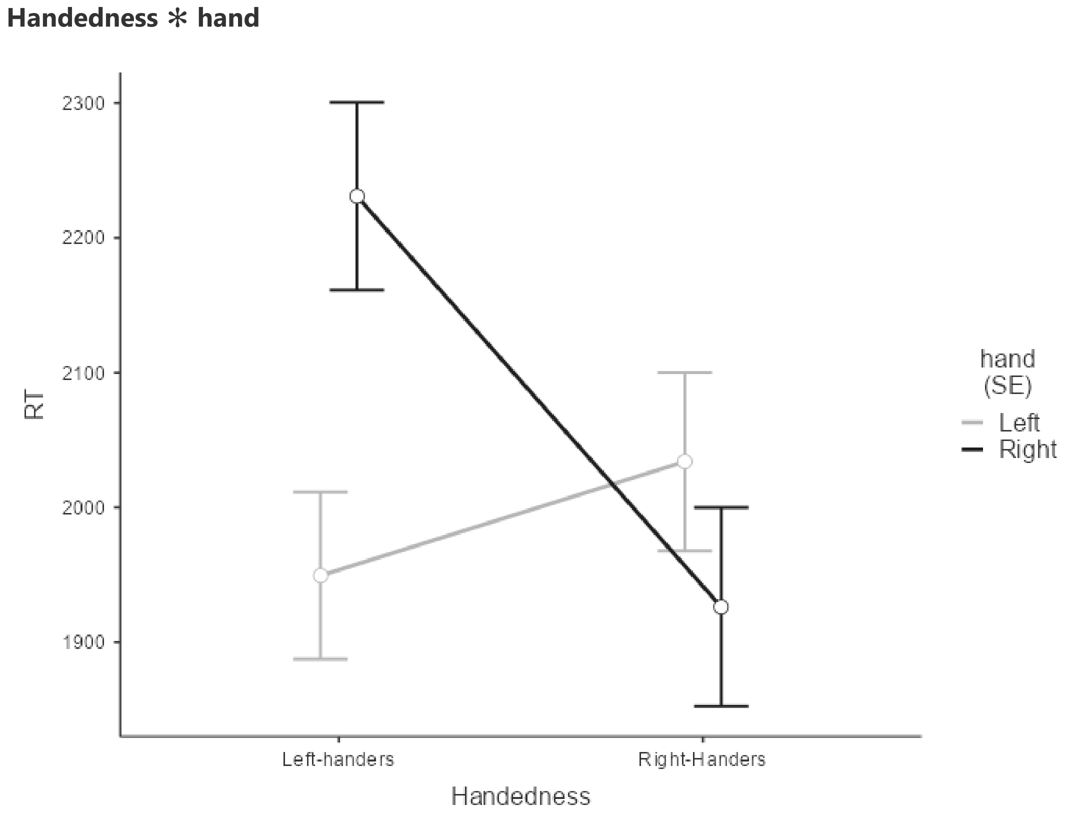

Figure SM3. RTs as function of the dominant hand: left-handers were faster with the left-dominant hand and right-handers were faster with the right-dominant hand (p < .001)

(A) (B)

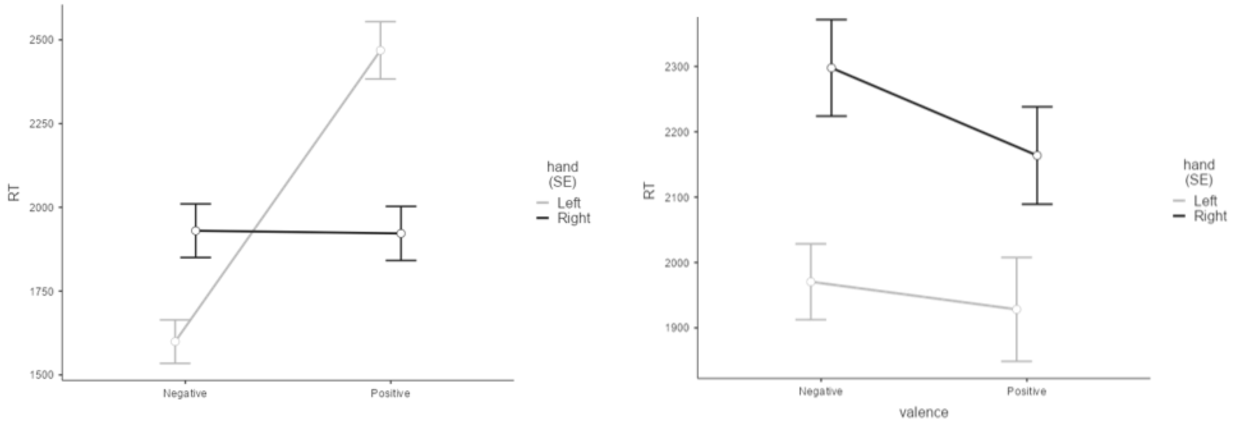

Figure SM 4. The Valence x Hand interaction is significant for right-handed participants (Panel A, p < .00) but not left-handed ones (Panel B, p = .24).

The combined analysis supported the previous results except for the Condition factor which did not reach a statistical difference to be significant with Handedness although, in the same model, the simple effect analysis is consistent with the separated result obtained for right-handed and left-handed participants. Specifically, these two groups, in the main text, respectively present a significant statistical effect (right-handers) and one smaller non-significant effect (left-handers) for the Condition factor. The simple effect analysis in the pooled analysis proves this, showing the same pattern.

2. Complete list of results

EXPERIMENT 1

Response time

Fixed Effects Omnibus Tests

	F	df	df (res)	p
valence	101.26367	1	23.0	< .001
condition	8.01548	1	23.0	0.009
hand	3.57670	1	23.0	0.071
valence ✻ condition	0.06499	1	1436.0	0.799
valence ✻ hand	148.62662	1	27.3	< .001
condition ✻ hand	2.93707	1	1436.0	0.087
valence ✻ condition ✻ hand	0.00492	1	1436.0	0.944

ANOVA for **Simple Effects of Valence**

Moderator				
**hand**	**F**	**Num df**	**Den df**	**p**
1 right	0.00200	1.00	26.7	0.966
2 left	176.56600	1.00	23.3	< .001

Parameter Estimates for simple effects of Valence

Moderator				95% Confidence Intervals				
**hand**	**Effect**	**Estimate**	**SE**	**Lower**	**Upper**	**df**	**t**	**p**
1 right	2 positive - 1 negative	8.84e-4	0.0203	-0.0408	0.0426	26.7	0.0436	0.966
2 left	2 positive - 1 negative	0.395	0.0297	0.3333	0.4562	23.3	13.2878	< .001

ANOVA for **Simple Effects of hand**

Moderator				
valence	F	Num df	Den df	p
1 negative	37.1	1.00	26.1	< .001
2 positive	47.5	1.00	23.1	< .001

Parameter Estimates for simple effects of hand

Moderator				95% Confidence Intervals				
valence	Effect	Estimate	SE	Lower	Upper	df	t	p
1 negative	2 left - 1 right	-0.148	0.0243	-0.198	-0.0980	26.1	-6.09	< .001
2 positive	2 left - 1 right	0.246	0.0357	0.172	0.3198	23.1	6.89	< .001

Valence evaluation

Fixed Effect Omnibus tests

	F	Num df	Den df	p
valence	9.7450	1	23.0	0.005
hand	0.0170	1	2611.0	0.896
condition	0.0418	1	23.0	0.840
valence ✻ hand	0.5443	1	2634.0	0.461
valence ✻ condition	0.0170	1	2634.0	0.896
hand ✻ condition	3.1660	1	2611.0	0.075
valence ✻ hand ✻ condition	0.1107	1	2611.0	0.739

Nota. Satterthwaite method for degrees of freedom

EXPERIMENT 2

Response time

Fixed Effects Omnibus Tests

	F	df	df (res)	p
valence	8.11	1	25.0	0.009
hand	21.03	1	25.0	< .001
condition	1.53	1	25.0	0.228
valence ✻ hand	2.10	1	2779.0	0.147

Fixed Effects Omnibus Tests

	F	df	df (res)	p
valence ✻ condition	1.46	1	2779.0	0.227
hand ✻ condition	1.43	1	25.0	0.244
valence ✻ hand ✻ condition	7.43e-4	1	2779.0	0.978

Valence evaluation

Fixed Effect Omnibus tests

	F	Num df	Den df	p
valence	0.0309	1	25.0	0.862
hand	0.0933	1	2829.0	0.760
Condition	7.4190	1	25.0	0.012
valence ✻ hand	0.7108	1	2829.0	0.399
valence ✻ Condition	1.5229	1	2829.0	0.217
hand ✻ Condition	1.1299	1	2829.0	0.288
valence ✻ hand ✻ Condition	0.0431	1	2829.0	0.835

Nota. Satterthwaite method for degrees of freedom
