# Supplementary material for: Exploring Body‐Specific Associations in Swipe Gestures: A Study on Hand Dominance and Emotional Valence
Source: Int J Psychol. 2026 Jan 5;61(1):e70154. doi: 10.1002/ijop.70154 (PMC12770064; doi:10.1002/ijop.70154)
Supplement: Supplementary file 2 — Data S2: ijop70154‐sup‐0002‐Supplementarytable.pdf. [file IJOP-61-e70154-s001.pdf]

Supplementary table

Descriptive statistics (mean and std. dev) of selected images

| Image | Category | Valence_mean | Valence_SD |
|---|---|---|---|
| p36 | Positive | 4.68627451 | 0.954202712 |
| p20 | Positive | 4.519607843 | 0.841149522 |
| p17 | Positive | 4.754901961 | 0.958921367 |
| p10 | Positive | 4.685185185 | 1.189110625 |
| p31 | Positive | 4.25 | 1.136686445 |
| p33 | Positive | 4.166666667 | 0.952125021 |
| p14 | Positive | 4.768518519 | 1.172954425 |
| p25 | Positive | 5 | 1.128683921 |
| p38 | Positive | 4.537037037 | 1.045023149 |
| p12 | Positive | 4.475247525 | 1.15407157 |
| p30 | Positive | 5 | 1.100854753 |
| p15 | Positive | 4.148068689 | 1.067795353 |
| p19 | Positive | 4.53955033 | 1.071410655 |
| p21 | Positive | 4.154408963 | 1.048623321 |
| n25 | Negative | 4.354709963 | 1.098513848 |
| n1 | Negative | 4.702873876 | 0.954863711 |
| n21 | Negative | 4.555600332 | 1.040623143 |
| n37 | Negative | 4.752414826 | 1.06007659 |
| n29 | Negative | 4.828138094 | 1.141000933 |
| n6 | Negative | 4.746675536 | 1.028283765 |
| n19 | Negative | 4.210827833 | 1.042535982 |
| n15 | Negative | 4.571414273 | 0.999610516 |
| n38 | Negative | 4.034107043 | 1.236767972 |
| n22 | Negative | 4.756925771 | 1.39792119 |
| n9 | Negative | 4.625851888 | 1.361177335 |
| n10 | Negative | 4.343596974 | 1.366778646 |
| n20 | Negative | 4.096741512 | 1.044022283 |
| n39 | Negative | 4.095721235 | 1.47210711 |
